# RSV Fusion: Time for a New Model

**DOI:** 10.3390/v5030873

**Published:** 2013-03-19

**Authors:** Peter Mastrangelo, Richard G. Hegele

**Affiliations:** Department of Laboratory Medicine and Pathobiology, University of Toronto, Ontario, M5S 1A8, Canada; E-Mail: peter.mastrangelo@utoronto.ca

**Keywords:** RSV, nucleolin, receptor

## Abstract

In this review we propose a partially hypothetical model of respiratory syncytial virus (RSV) binding and entry to the cell that includes the recently discovered RSV receptor nucleolin, in an attempt to stimulate further inquiry in this research area. RSV binding and entry is likely to be a two-step process, the first involving the attachment of the virus to the cell membrane, which may be enhanced by electrostatic interactions with cellular glycoproteins/heparin and the viral G protein, and the second involving fusion to the cell membrane mediated by the viral F protein and a specific cellular fusion receptor. With our recent discovery of nucleolin as a functional fusion receptor for RSV, comes the possibility of a number of new approaches to the development of novel strategies for RSV prophylaxis and therapy, as well as raising some new questions concerning the pathobiology of RSV infection and tropism.

## 1. Introduction

Human respiratory syncytial virus (RSV) is found ubiquitously and a major cause of acute lower respiratory tract infections in children leading to hospitalization and occasionally death [1]. RSV also causes disease in adults, particularly in the elderly and in the immunocompromised [2]. Treatment and prophylaxis, given primarily to infants, are limited to Ribavirin and Palivizumab, respectively [2]. RSV is a negative-polarity, enveloped, single-stranded RNA virus from the Paramyxoviridae family. The viral genome encodes 11 proteins, three of which, small hydrophobic (SH), glycoprotein (G) and fusion (F) contribute to the viral coat. In spite of the fact that the virus was discovered in 1956, a safe, effective vaccine for RSV has remained elusive [3]. 

In this article we discuss aspects related to the discovery of nucleolin as a functional fusion receptor for RSV and propose a revised model for RSV fusion at the cell surface that incorporates nucleolin. Also, we discuss the implications this discovery has on the pathobiology of RSV infections and the development of novel prophylactic and therapeutic strategies.

## 2. Viral Envelope Proteins and Fusion

In order to understand attachment and fusion/entry into cells it is crucial to determine which viral envelope protein(s) are involved and what are their corresponding cellular ligands. The RSV G protein is a heterogeneous glycoprotein that defines the two subtypes of RSV (A and B). Besides its role in attachment of virus to the cell surface, the RSV G protein also helps the virus to elude the host immune system by mimicking cellular cytokines and through shedding [4]. Fusion of the virus to the cell membrane as well as the formation of syncytia, the characteristic cytopathic effect of RSV, is mediated by the viral F protein. The F protein unlike the G protein is homologous to both subtypes of RSV. SH, the remaining viral envelope protein, is not required for RSV attachment or entry [5].

As will be discussed in further detail below, we propose that RSV binding and entry into cells is likely to be at least a two-step process: first, attachment of the virus to the cell membrane mediated by electrostatic interactions with cellular glycoproteins/heparin and the viral G protein, and secondly, fusion to the cell membrane mediated by the viral F protein and a specific cellular fusion receptor. 

## 3. Defining a Functional RSV Receptor

Molecules proposed to be the “RSV receptor” include intercellular adhesion molecule (ICAM)-1 [6], heparin [7], annexin II [8], toll-like receptor (TLR) 4 [9] and fractalkine (CX3CL1) receptor, CX3CR1 [10]. Prior to the discovery of nucleolin as a functional receptor of RSV, no candidate receptor molecule met all of the following criteria of being a functional receptor, including: (i) decreased infection through antibody neutralization, competition with soluble candidate receptor molecule or decreased receptor expression through RNA interference; (ii) increased infection of non-permissive cells after ectopic expression of the candidate receptor molecule on the cell surface [11]. Despite a lack of definitive validation of these various candidate receptor molecules by all of the above criteria to date, one or more of them could function as co-receptors or co-factors for RSV and nucleolin. 

Binding of viral G protein in an electrostatic fashion to the cell surface may be the first step in efficient viral attachment prior to fusion via nucleolin. Heparin, for example, binds the RSV G protein [7] on HEp-2 cells, however it turns out that human airway epithelium does not express heparin on the apical surface, the site of RSV attachment and cellular entry [12]. Thus the binding to heparin by the G protein may simply serve to demonstrate that the G protein has a general affinity for negatively charged carbohydrates on the cell surface. Further, mutant forms of RSV that lack the G protein (RSVΔG) are capable of causing productive infections, albeit at much lower efficiency than wild-type RSV [13]. 

The RSV F protein is necessary for infection, as mutant RSV lacking F protein (RSVΔF) cannot infect cells on its own but rather requires a helper virus to gain entry into cells [14]. In contrast to RSV G, the F protein has a list of much more specific protein interactions. One example of such a protein interaction is RSV-F protein with intercellular adhesion molecule (ICAM)-1 expressed on the cell surface. Although it has not been definitively shown to be essential for viral fusion, ICAM-1 has been reported to bind the F protein and may as such still have a role in fusion [6]. Importantly, despite the apparently greater specificity of RSV F-protein interactions *vs*. RSV G, one must exercise caution over RSV F-protein interactions, as Toll-like receptor (TLR) 4 binds the purified RSV F protein, yet has no effect on viral infectivity in cell culture [9].

## 4. Nucleolin: A Functional Fusion Receptor of RSV [15]

### 4.1. Identifying Nucleolin as a Ligand of Intact RSV

To search for candidate RSV receptor molecules, we performed a number of preliminary experiments in which cell cultures from numerous mammalian species were treated with enzymes of cell surface components (protein, lipid, carbohydrate) and effects of enzyme pre-treatment on subsequent RSV infection were quantified. Results showed that pre-treatment of cells with trypsin, a protease, resulted in lower RSV infection than occurred in cells that did not undergo enzyme treatment, without affecting cell viability. These findings provided the rationale to use a Virus Overlay Protein Binding Assay (VOPBA) to identify candidate RSV receptor molecules [16]. VOPBA is essentially a modified Western blot where the virus substitutes for the primary antibody and is then detected with a virus-specific secondary. In protein extracts from various mammalian cell lines and numerous RSV isolates (including both laboratory-adapted and community strains of RSV A and RSV B), we reproducibly identified a VOPBA signal of approximately 100 kDa. As one would expect, a number of “hits” were obtained by mass spectrometry of this 100 kDa band, but nucleolin was the most consistent, being found in every extract and viral isolate tested, and it also satisfied the requirement of being found at the cell surface [17,18]. As noted above, RSV F and G are the envelope proteins primarily involved in cell surface binding. To determine if nucleolin binds to RSV F or G, immunoprecipitations (IPs) were performed. Nucleolin co-precipitated only with the viral F protein in every instance tested. Addition of excess heparin to the IPs did not interfere with the nucleolin-protein F interactions in IPs although we also saw, as have others, a decrease in RSV infection *in vitro* [13].

The biological plausibility of RSV-nucleolin interaction in infection was confirmed *in vitro* and *in vivo* through a series of experiments that included: visualization of RSV-nucleolin co-localization on the cell surface by use of confocal fluorescence microscopy; decreased RSV infection of cells pre-treated with nucleolin-specific antibody and when cellular nucleolin expression was silenced by use of RNA interference, or when virus was incubated with soluble recombinant nucleolin prior to being added to cell cultures; increased RSV infection of a non-permissive cell type (Sf9) [36] that had been transfected with the human nucleolin gene and which showed ectopic expression of human nucleolin protein on the cell surface; decreased RSV infection of mouse lung, in animals that were pre-treated with short-interfering RNA of mouse nucleolin, delivered intranasally prior to RSV challenge.

### 4.2. Nucleolin: Brief Overview

First described in 1973, nucleolin is a multifunction protein that is found throughout the cell but it is primarily localized within the nucleolus, contributing up to 10% of the total protein in that compartment [19,20]. Although its predicted molecular weight is 77-78 kDa (depending on the species), its relative molecular mobility in SDS-PAGE is 100-110 kDa [21], due to highly phosphorylated amino acids of the N-terminus [22]. Nucleolin has been shown to be more stable in proliferating cells due to inhibition of an auto-proteolytic activity more prominently found in quiescent cells [23]. Nucleolin is involved in diverse biological processes including cell proliferation, growth, cytokinesis, replication, embryogenesis and nucleogenesis and is considered necessary for cell survival and proliferation [24].

Nucleolin has a very high degree of evolutionary conservation [25] and can be divided into three structural/functional domains: (i) multiple acidic stretches in the N-terminus; (ii) multiple RNA recognition motifs (RRMs) in the central portion and, (iii) a glycine/arginine rich domain in the C-terminal portion [21]. Although nucleolin is typically thought of first and foremost as an intranuclear protein [25], there is abundant evidence that it can also be found within the cytoplasm and on the cell surface and may play the role of a “molecular shuttle” between these compartments [24,26]. Nucleolin has a bipartite nuclear localization signal whose function is regulated by phosphorylation [27]. The actin cytoskeleton modulates the entry of substances via nucleolin into the cytoplasm [28]. The half-life of cell surface nucleolin is less than one hour and its expression is very sensitive to inhibition of transcription/translation, unlike nuclear nucleolin that has a half-life greater than eight hours [26].

In contrast to other cell surface proteins, nucleolin does not have a transmembrane domain or a glycosylphosphatidyl-inositol (GPI) anchor [26]; instead, nucleolin exists on the cell surface as part of a 500 kDa protein complex that includes other membrane proteins [29]. Nucleolin functions as a receptor for a number of different molecules including DNA nanoparticles [30], apoB/E-containing lipoproteins, laminin-1, viruses (see below) [24] and bacteria [31,32].

Nucleolin also plays a role in viral replication and intracellular trafficking of viral components. For example nucleolin is required for HSV-1 DNA replication [33] and also for trafficking of the viral protein US11 out of the nucleus [34]. It also has been shown to bind the RNA-dependent RNA polymerase of HCV (NS5B) [35]. In HCMV nucleolin helps to maintain the architecture of viral replication compartments [36]. Similarly, nucleolin has been shown to bind the 3’ untranslated regions and protease-polymerase NS6/7 of feline calicivirus again having a role in viral replication [37]. That these roles in viral replication and trafficking are connected to nucleolin’s role as a viral receptor has yet to be determined.

## 5. A New Model for RSV Fusion/Entry

In light of our findings of expression of cell surface nucleolin being sufficient for RSV infection, a revised model for RSV fusion can be generated. The present models for RSV fusion are in part inferred by analogy to data obtained for other enveloped viruses, particularly influenza virus [38,39]. The RSV F protein initially exists in a pre-fusion state that then undergoes conformational changes to a post-fusion form upon binding to receptor molecules expressed on the cell surface [31]. Current models reflect the fact that comparatively little is known about receptors or other factors involved in RSV fusion, and what triggers the conformational changes in the RSV F protein required for fusion. What seems evident from the results of our experiments is that nucleolin can allow this event to occur. We propose a model were nucleolin binds the pre-fusion conformation of the F protein possibly priming transition to the “extended” intermediate conformation [39]. Evidence that it is the pre-fusion form of RSV F that binds nucleolin is provided by results of our VOPBA experiments, in which live virus was shown to specifically bind nucleolin immobilized on a membrane in the absence of any cells [13]. It follows that this interaction would be with the pre-fusion conformation of the F protein, as “triggering” has not yet occurred. Furthermore, in so-called “competition” experiments in which RSV was incubated with soluble, recombinant nucleolin prior to being added to cell cultures, this state involved exogenous nucleolin saturating F protein binding sites in a pre-fusion state, since there were no cells present during this incubation step.

**Figure 1:**
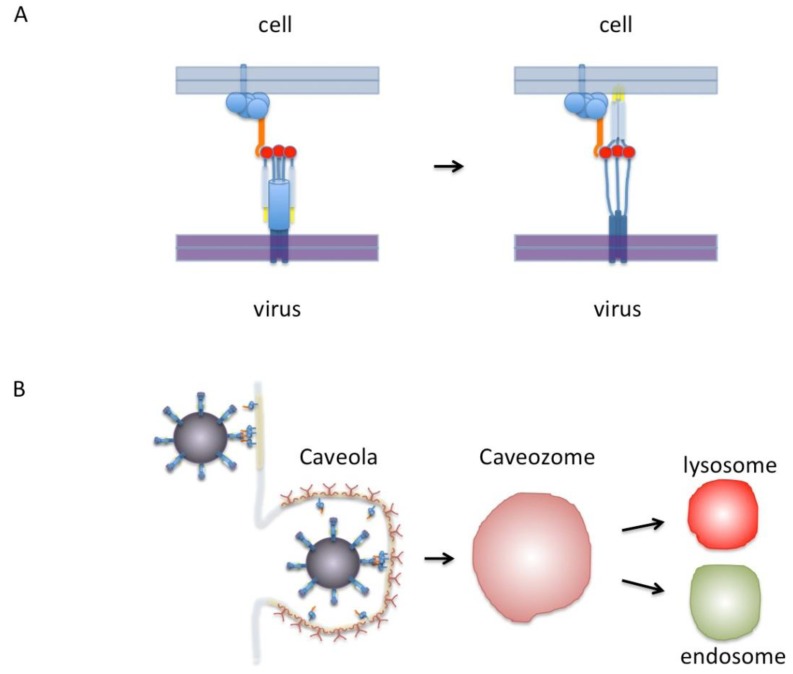
(A) Model of RSV F-protein binding nucleolin. On the left, the F-protein is shown in its trimeric pre-fusion conformation. The red circles are putative nucleolin binding sites. Nucleolin is shown in orange as part of a protein complex that includes proteins anchored to the membrane by either a transmembrane domain or a GPI anchor. Only one nucleolin molecule is shown binding the F-protein trimer for clarity but in this model as many as three could bind at once. On the right the F-protein is shown in the “extended” conformation with fusion peptides (yellow) inserted into the cell membrane. After this step virus-cell membrane fusion would proceed without nucleolin. **(B)** Diagram of virus binding to the cell surface. Indicated in light yellow are lipid-rich domains/rafts. The virus is shown in a dark magenta covered with F-protein binding to nucleolin that is preferentially located in lipid-rich rafts or caveolae. A caveola is shown covered with caveolin (dark red). This in turn can enter the cell to form a caveozome and join the endosomal or lysosomal pathway. Our proposed model leaves open the possibilities that viral fusion may occur at the cell surface or in a caveozome/endosome/lysosome. Another possibility (not shown) is that virus enters via clathirin-coated pits (see text).

In our proposed model (Figure 1A), RSV binds cell surface nucleolin via the trimeric pre-fusion form of the F protein. Nucleolin is shown as part of a protein complex which includes either membrane proteins or glycosylphosphatidylinositol (GPI)-linked proteins that tether it to the cell surface. We postulate this binding event triggers the re-folding of the F protein into its extended conformation and once this has occurred, fusion can continue without further need of nucleolin. In this model as many as three molecules of nucleolin can bind one RSV F protein pre-fusion trimer. Refolding of the fusion protein from a pre-fusion to post-fusion conformation is irreversible making the temporal and spatial triggering of this event critical [38]. A fusion receptor like nucleolin helps to ensure that the optimal conditions for viral fusion are met.

Cell surface nucleolin has been found in association with lipid-rich rafts upon anchorage of HIV to permissive cells [40] and efficient RSV infection of human lung epithelial cells requires intact lipid-rich rafts [41]. Nucleolin-mediated trafficking of DNA nanoparticles is lipid raft dependent and nucleolin colocalizes with and co-immunoprecipitates with the raft protein, flotillin [42]. Thus nucleolin may serve to bring RSV in contact to raft domains that may themselves be preferred areas for virus fusion. Alternatively, RSV may be brought into the cell via caveolae or endosomes and triggering/fusion might occur in one of these intracellular compartments. Uptake of virus by bovine dendritic cells has been shown to involve caveolae and RSV colocalizes with caveolin in these cells [43]. However, unlike the case of influenza and Semliki forest virus (SFV) [44], RSV F protein does not appear to be triggered by pH change that occurs during the acidification of lysosomes [45,46]. Interestingly, it has been reported that the internalization of nucleolin on ligand binding is a calcium-dependent process [26] and it has been known for some years that RSV infection is also a calcium-dependent process [47]. On the other hand, there is a body of evidence that supports the idea of clatherin-mediated endocytosis being necessary for RSV infection [48]. Similarly, there are publications that show that nucleolin is internalized after ligand binding (*i.e.*, endostatin) via a clatherin-mediated pathway [49]. It seems possible that RSV and nucleolin may use both types of endosomal pathways in different circumstances however this still remains to be resolved. 

As depicted in Figure 1B, RSV binding to the cell surface is shown incorporating lipid-rich rafts or caveolae. Based on available evidence, including analogies to other pathogens, there exist a number of possibilities by which this can occur and how nucleolin may be involved. For example, nucleolin may either be located within lipid rafts or may become associated with these rafts after binding to the virus. Moreover, lipid raft formation may even be induced by the aggregation of nucleolin around the viral fusion protein. At this point either fusion occurs immediately or the virus is brought into the cell possibly via a caveosome and fusion is triggered later on. Overall, the model we show here, while incomplete, provides a framework with which these various possibilities can be tested experimentally.

## 6. Future Directions

### 6.1. Targeting the Host in RSV Prophylaxis and Therapy?

As has been demonstrated for Palivizumab [50], targeting RSV as a strategy for patient management has limitations, since resistant viral strains can arise through random genetic mutation and selective pressure. An alternative strategy is to target the host rather than the virus, the human genome being much more stable. This is not as radical an idea as it may sound if one considers that when immunizing a patient against infection the goal is “conversion” of the host to a virus-resistant state –in effect the host, not the virus, is being targeted. The challenge is to achieve an adequate anti-viral state in the host, and to avoid undesired off-target effects or other toxicity. 

### 6.2. Nucleolin as an Anti-RSV Target

Nucleolin has a rapid half-life on the cell surface: exposure of cells to nucleolin-specific antibody results in rapid internalization of the nucleolin-antibody complex and replacement with “fresh” nucleolin [51]. The rapid turnover of the cell surface nucleolin may be advantageous in the context of designing RSV prophylaxis and/or therapy since it is unlikely there would be any long-lasting disruption of its normal functions. Targeting cell surface nucleolin with the guanine-rich oligonucleotide (GRO), AS1411 [52], is being evaluated in the therapy a variety of cancers in human clinical trials. Other nucleolin-binding compounds (*e.g.*, midkine, pleiotropin, lactoferrin, pseudopeptide HB-19) have been described and in some cases, their safety profiles have already been established in humans. An important practical consideration is that, in contrast to use in chronic diseases such as cancer, nucleolin-binding compounds used for either prophylaxis or therapy of RSV infections (*e.g.*, as a nasal spray formulation) would be administered for relatively short intervals and thus could avoid the development of some of the undesired drug-related effects associated with longer term use. 

### 6.3. RSV-Nucleolin Interaction Domain(s)

Detailed inspection of the primary amino acid sequence of nucleolin does not provide any apparent clues about potential interaction domain(s) with RSV, although one might reasonably excludethe RRMs found in the central portion because these domains are known to be nucleic acid binding motifs [21]. While binding cell surface nucleolin with a specific compound that targets nucleolin and causing it to internalize may be sufficient to make it unavailable for binding to virus, determining the interaction domains of nucleolin and the RSV F protein will be essential for the rational design of small molecule inhibitors. Also, if cell surface nucleolin is part of a larger protein complex [29] that functions to tether it to the cell surface, one could potentially target the other proteins in this complex in order to destabilize it.

One potential site of RSV-F protein interaction with nucleolin may occur with the “head” around amino acids 429-437. This region of the protein binds mAb 19 and mAb 20, both monoclonal antibodies known to inhibit RSV fusion [53,54,55]. However, such associations based on antibody binding epitopes need to be interpreted cautiously as interference via an antibody may only require that it bind near enough to the interaction domain to be an effective at neutralization.

### 6.4. Nucleolin and RSV Tropism

**Figure 2:**
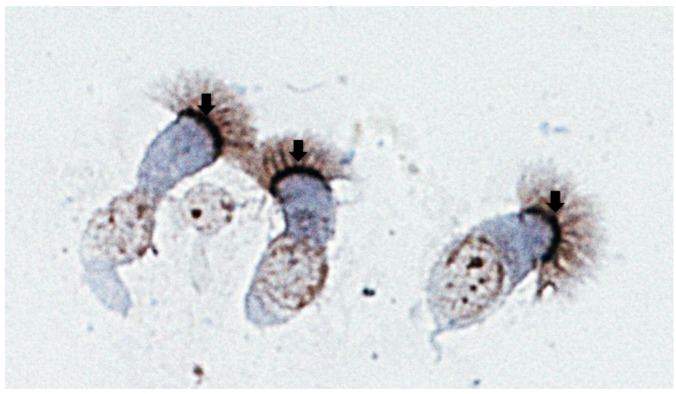
Human airway epithelial cells from bronchial brushings stained with a rabbit polyclonal antibody against human nucleolin (H-250, Santa Cruz Biotechnology Inc., Santa Cruz , CA). Note the positive nucleolin immunostaining (dark brown) at the apical surface of airway epithelial cells (arrows).

Because nucleolin is an essential protein found in all cells, it does not by itself account for the apparent restricted tropism of RSV to the respiratory tract and while it is clearly expressed on the cell surface of airway epithelium, the major target for RSV infection, it has not yet been determined if this is the case for other cell types *in vivo* (Figure 2). However, this situation is not unique to RSV, as other viral receptors are widely expressed in various cells and tissues, while tropism is restricted [56]. For example, the cellular receptor of Hepatitis A virus, HAVcr-1, is expressed in various cells within in the gastrointestinal tract, salivary glands, kidney, spleen and lymph nodes, yet clinically the virus shows marked tropism to the liver [57]. We speculate that the tropism of RSV for the respiratory tract could involve a combination of the local environment/context and possibly the involvement of more than one receptor/co-receptor, including other molecules postulated to be RSV receptors indicated above. In this context, the viral G protein may be an important determinant of RSV’s tropism to the respiratory tract, in terms of the role of RSV G in initial attachment and enabling the virus to recognize nucleolin expressed on the cell surface. Given that the efficiency of RSV infection is markedly decreased in RSVΔG compared to wild-type virus, a role for RSV G as a determinant of tropism is likely.

## 7. Concluding Remarks

Our discovery of nucleolin as a functional RSV fusion receptor *in vitro* and *in vivo* increases our understanding of the pathobiology of RSV infections, and presents a number of possibilities for novel interventions, including targeting RSV fusion from a “host” perspective. Although the mechanisms of RSV fusion require further study, we present a model of RSV fusion that has a number of testable assumptions and could lead to a deeper understanding of the events underlying RSV fusion and entry into cells.

## Supplementary Files

Supplementary File 1 Supplementary Information (JPG, 109 KB)
