# Supplementary figures and images for: RSV Fusion: Time for a New Model
Source: Viruses. 2013 Mar 19;5(3):873–85. doi: 10.3390/v5030873 (PMC3705301; doi:10.3390/v5030873)

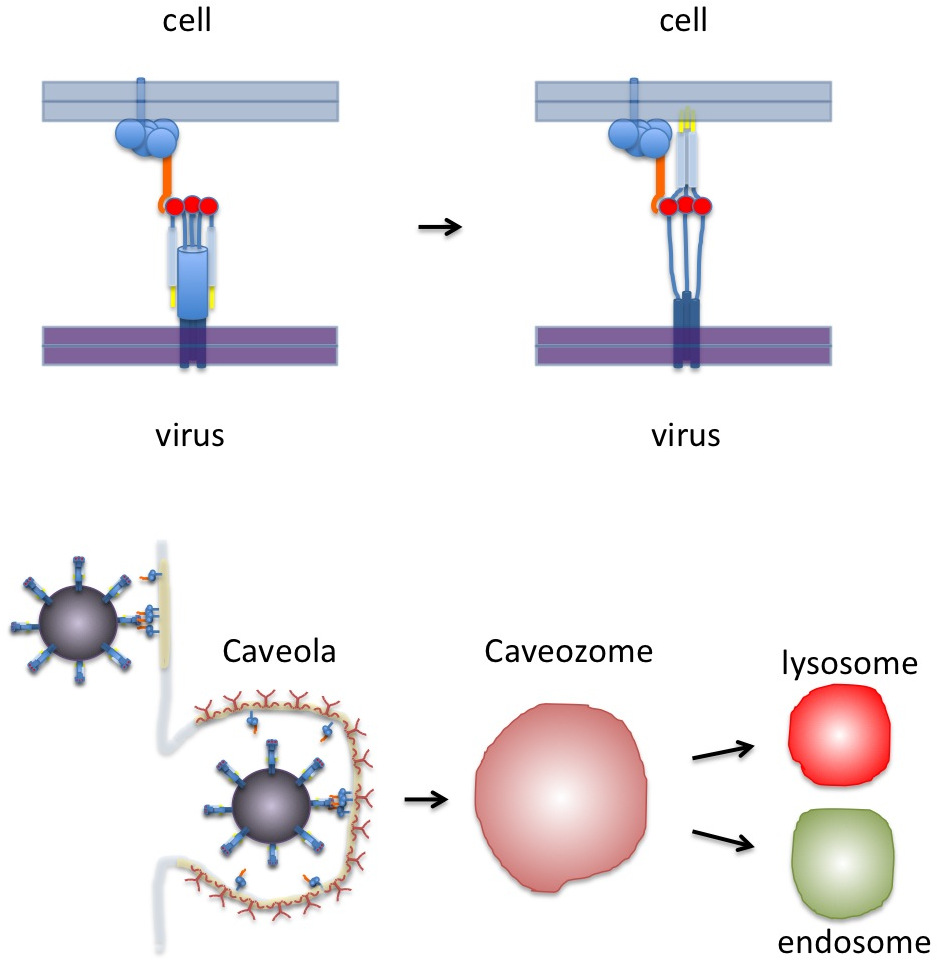

Supplement: Supplementary File 1 — Supplementary Information (JPG, 109 KB) [file viruses-05-00873-s001.jpg]
